# Knowledge, attitudes and practices regarding children with ICU-acquired weakness in pediatric intensive care unit among chinese medical staff: a cross-sectional survey

**DOI:** 10.1186/s12912-023-01304-x

**Published:** 2023-05-15

**Authors:** Di Huang, Weiwei Zhang, Weisi Peng, Yi Fan, Xin He, Ruirui Xing, XuDong Yan, Sijia Zhou, YueMing Peng, WeiXiang Luo

**Affiliations:** 1 Shenzhen institute of respiratory Diseases, Shenzhen People’s Hospital (The Second Clinical Medical College, Jinan University; The First Affiliated Hospital, Southern University of Science and Technology), Shenzhen, 518020 Guangdong China; 2 Department of nursing, Shenzhen People’s Hospital (The Second Clinical Medical College, Jinan University; The First Affiliated Hospital, Southern University of Science and Technology), Shenzhen, 518020 Guangdong China; 3 Department of PICU, Shenzhen People’s Hospital (The Second Clinical Medical College, Jinan University; The First Affiliated Hospital, Southern University of Science and Technology), Shenzhen, 518020 Guangdong China

**Keywords:** Health Knowledge, Attitudes, Practice, Muscle weaknessSurveys and questionnaires, Pediatric Intensive Care Units

## Abstract

**Background:**

ICU-AW (Intensive Care Unit Acquired Weakness) is characterized by significant muscle weakness and can be caused by a variety of factors, including immobility, medication use, and underlying medical conditions.ICU-AW can affect critically ill children who have been hospitalized in the PICU for an extended period of time.The knowledge, attitude and practice level of ICU-AW of PICU medical staff directly affect the treatment of critically ill children with ICU-AW.The aim to this study was to explore the knowledge, attitudes, and practices of Chinese medical staff regarding critically ill children with intensive care unit-acquired weakness (ICU-AW) and related factors.

**Methods:**

A Knowledge, Attitudes, and Practices (KAP) Questionnaire regarding critically ill children with ICU-AW was distributed to a stratified sample of 530 pediatric intensive care unit (PICU) healthcare workers. The questionnaire consisted of 31 items—with scores of 45, 40, and 40 for each dimension and a total score of 125.

**Results:**

The mean total score of Chinese PICU healthcare workers for the KAP questionnaire regarding children with ICU-AW was 87.36 ± 14.241 (53–121), with mean total knowledge, attitudes, and practices scores of 30.35 ± 6.317, 30.46 ± 5.632, and 26.54 ± 6.454, respectively. The population distribution indicated that 50.56%, 46.04%, and 3.4% of healthcare workers had poor, average, and good scores, respectively. Multiple linear regression showed that gender, education, and hospital level classification influenced the KAP level of PICU healthcare workers regarding critically ill children with ICU-AW.

**Conclusions:**

Overall, PICU healthcare workers in China have an average KAP level about ICU-AW, and the gender and education level of PICU healthcare workers, as well as the classification of hospitals where they work, predict the KAP status of healthcare workers regarding children with ICU-AW. Therefore, healthcare leaders should plan and develop specific training programs to improve the KAP level of PICU healthcare workers.

**Supplementary Information:**

The online version contains supplementary material available at 10.1186/s12912-023-01304-x.

## Introduction

ICU-acquired weakness is defined as unexplained weakness in a critically ill patient during hospitalization in an intensive care unit (ICU) [1] and includes critical illness polyneuropathy (CIP), critical illness myopathy (CIM), or both. ICU-acquired weakness (ICU-AW) is characterized clinically by diffuse symmetrical weakness of the limbs, decreased muscle strength, weakened reflexes, and muscle atrophy, and most involvement is of the lower limb and respiratory muscles [2]. After studies confirmed that ICU-acquired frailty was significantly related to a decline in body function and an increase in mortality after discharge, ICU-acquired frailty was given attention and further explored by the public [3]. The pathogenesis of ICU-acquired frailty remains unknown, and intervention against the risk factors of acquired weakness is currently one of the most feasible ways to prevent and treat the disease. The main risk factors for the onset of ICU-AW are immobilization, hyperglycemia, multiple organ failure, systemic inflammatory response, and drug treatment. When adults undergo mechanical ventilation for > 4–7 days, the prevalence of ICU-AW is 33–82% [4]. The prevalence of ICU-AW can be as high as 100% in patients with hyperemia or multiple organ failure [5].

In contrast, reports of CIP and CIM in pediatric patients are limited. In an early study, Banwell et al. reported that the incidence of CIP/CIM among 830 pediatric ICU patients was only 1.7%, which was lower than that in adults [6]. However, in a subsequent study, Vondracek et al. found electrophysiological abnormalities among two in five critically ill children, confirming that CIP/CIM is more common than previously suggested [7]. In addition to individual cases, a study by Thabet et al. on 105 critically ill children found that 35 patients developed changes associated with CIP/CIM [8]. However, in a prospective study by Kasinathan et al., the incidence of ICU-AW among children who underwent serial electrophysiological assessments was extremely low, with only 2 of the 97 enrolled patients diagnosed with ICU-AW [9]. The above survey results show that the incidence of ICU-AW in critically ill children varies, possibly due to the lack of awareness of this disease among pediatric ICU (PICU) medical staff and the difficulty in evaluating clinical symptoms in critically ill children. Most symptoms related to ICU-AW were detected in critically ill children after being weaned from mechanical ventilation. In additional,The incidence of ICU-AW may differ depending on the age of the child. For example, younger children may be less likely to develop ICU-AW due to the relative preservation of muscle mass compared to older children[10]. Before developing interventions for ICU-AW in critically ill children, the knowledge, attitudes, and practices of PICU medical staff must be investigated.

No Chinese or foreign data currently exists illustrating the knowledge, attitudes, and practices of PICU medical staff. Therefore, this study investigates and analyzes the knowledge, attitudes, and practices of PICU medical staff across China on critically ill children with ICU-AW; analyzes its influencing factors; and provides relevant evidence for later formulation of intervention measures for children with ICU-AW.

## Materials and methods

### Design

This was a national cross-sectional survey study conducted from January 1, 2021, to February 28, 2022.

### Setting and participants

The medical staff included were clinicians, clinical nurses, pharmacists, rehabilitators, physiotherapists, researchers, educators, and PICU managers [11]. A multi-layer cluster random sampling method was used to obtain the final sample. Hospitals were classified into “three levels and six grades” according to the Measures for the Management of Hospital Grading of China and are divided into specialty hospitals and general hospitals. According to the database of the National Care Commission of China in December 2017, there were 1151 Level 3 hospitals, including 705 and 198 Level 3 Grade A and B hospitals, respectively. Most PICUs in China are currently located in Level 3 hospitals due to the numerous requirements for PICU setup. As such, this study sampled 15, 13, 6, and 4 Level 3 Grade A general hospitals, Grade A children’s hospitals, Grade B general hospitals, and Grade B children’s hospitals with PICUs from each of the geographical locations (eastern, central, and western regions) in China, respectively. SPSS software was used to number each hospital, and the 38 hospitals randomly selected were contacted by WeChat or email to inform the hospital managers or PICU unit management directors of the purpose and procedure of the questionnaire and to obtain their consent and assistance. All enrolled medical staff from the PICU of each hospital were assigned with numbers, and 25% of the enrolled personnel from each selected hospital were assigned randomly proportionate to the allocation ratio. Subsequently, the unit manager of each hospital’s PICU invited the PICU healthcare staff to participate in this study. The sample size was chosen according to the Kendall sample size method [12], which requires a sample size of five to ten times the number of questionnaire items. Given that the questionnaire in this study consisted of 31 items, 310 respondents were needed to meet the sample size requirement.We considered possible 10–20% missing, and the final sample size required a minimum was on 372. Inclusion criteria included: (1) healthcare workers with a professional license to practice (only nurses and doctors); (2) staff working in the PICU for at least one year. Exclusion criteria included: (1) residents with a qualification certificate; graduate students in rotational training in non-critical care; and (2) staff not on duty due to illness, maternity leave, or personal reasons. (3) Those who have participated in the relevant training of ICU-AW.

### Measurements

#### General data questionnaire

The demographics and institutional information consisted of gender, age, years of service, position, education, position level, tenure, city level, number of PICU beds, and hospital level classification. In this study, chinese medical staff professionals’hierarchies designations were classified as Level 1-Level 5(See Table 1) [13]. In the present study, cities were divided into first (Level 1), second (Level 2), and third (Level 3) tier cities, according to the 2019 Chinese City-tier New Classification by CBN [14]. The classification standard of hospitals is an index for evaluating hospital qualifications in my country based on hospital scale, scientific research direction, talent and technical strength, and medical hardware equipment.According to the “Hospital Classification Management Standard”, the hospital has been reviewed and determined as three level in view of the hospital’s scale, facilities and equipment. Furthermore each level is subdivided into A, B, and C based on hospital’s management and service quality (See Table 2) [15].

**Table 1:** General Characteristics of participants(n = 530)

Variables	N(%)	Variables	N(%)
**Gender**		**Education**	
Male	51(9.6)	Junior College	65(12.3)
Female	479(90.4)	Undergraduate	401(75.7)
**Age**		Master	54(10.2)
18–25	78(14.7)	Doctor	10(1.9)
26–30	147(27.7)	**Years of work**	
31–40	236(44.5)	＜3	70(13.2)
41–50	55(10.4)	3–5	73(13.8)
51–60	14(2.6)	5–10	179(33.8)
**Position**		11–15	119(22.5)
Doctor	102(19.2)	16–20	36(6.8)
Nurses	428(80.8)	＞20	53(10)
**Professional TiTle**		**City**	
Level 1	113(21.3)	Level 1	326(61.5)
Level 2	175(33)	Level 2	102(19.2)
Level 3	189(35.7)	Level 3	102(19.2)
Level 4	40(7.5)	**Bed Range of PICU**	
Level 5	13(2.5)	＜10	126(23.8)
**Position Title**		11–15	143(27)
physician manager	10(1.9)	16–20	135(25.5)
nurse manager	27(5.1)	＞20	120(22.6)
Advanced Practice Nurse	15(2.8)	**Hospital Type**	
Clinical Teaching Supervisor	13(2.5)	Level 3 Grade A general hospitals	366(69.1)
Nurses	369(69.6)	Level 3 Grade A Children’s hospitals	141(26.6)
Doctor	87(16.5)	Level 3 Grade B general hospitals	3(0.6)
Research assistant	2(0.4)	Level 3 Grade B Children’s hospitals	9(1.7)
other	7(1.3)	Level 2 Grade A Children’s hospitals	11(2.1)

**Table 2:** Response to statements regarding knowledge of PICU Chinese Medical Staff on Children with ICU-AW (n%)

Knowledge Statement	Mean ± SD	Do not know	Generally understand	Well understand
1.Do you know the related concepts of ICU-AW?	1.68 ± 0.791	277(52.3)	145(27.4)	108(20.4)
2.Do you know the clinical manifestations of ICU-AW?	1.62 ± 0.731	279(52.6)	172(32.5)	79(14.9)
3.Do you know how to diagnose ICU-AW?	1.45 ± 0.626	328(61.9)	164(30.9)	38(7.2)
4.Do you know how to evaluate ICU-AW patients?	1.50 ± 0.643	310(58.5)	177(33.4)	43(8.1)
5.Do you know the risk factors for ICU-AW?	1.58 ± 0.688	281(53.0)	188(35.5)	61(11.5)
6.Do you know the preventive measures of ICU-AW?	1.60 ± 0.703	279(52.6)	184(34.7)	67(12.6)
7.Did you know that critically ill children could also develop ICU-AW?	1.75 ± 0.805	253(47.7)	155(29.2)	122(23.0)
8.ICU-AW symptoms are muscle weakness with no clear cause in critically ill patients, clinically manifested as difficulty in weaning, paresis or quadriplegia, decreased reflexes, and muscle atrophy.	2.42 ± 0.592	28(5.3)	251(47.4)	251(47.4)
9.ICU-AW includes polyneuropathy in critically ill patients, myopathy in critically ill patients, and critical neuromuscular diseases.	2.41 ± 0.593	29(5.5)	256(48.3)	245(46.2)
10.The diagnosis of ICU-AW is mainly determined by the Medical Research Council Score (MRC-score).	2.30 ± 0.562	28(5.3)	315(59.4)	187(35.3)
11.Does the MRC-score use the Oxford Muscle Strength Scale to evaluate the six major muscle groups of the body?	2.32 ± 0.532	17(3.2)	325(61.3)	188(35.5)
12.ICU-AW not only prolongs the hospital stay and increases medical costs, but also reduces the patient’s ability to live and survive.	2.50 ± 0.558	16(3.0)	232(43.8)	282(53.2)
13. Braking may be an important risk factor for ICU-AW.	2.40 ± 0.556	18(3.4)	281(53.0)	231(43.6)
14. Early mobilization of ICU patients is the most effective intervention to prevent or mitigate ICU-AW in patients.	2.51 ± 0.533	9(1.7)	243(45.8)	278(52.5)
15. Standard insulin therapy can reduce the incidence and duration of neuromuscular complications, thereby reducing ICU-AW.	2.29 ± 0.522	17(3.2)	340(64.2)	173(32.6)

#### The knowledge, attitudes, and practices (KAP) questionnaire regarding children with ICU-AW

Guided by the knowledge-attitude-practice theoretical model, the questionnaire regarding critically ill Chinese patients with ICU-AW was combined with a literature review and group discussions to determine the conceptual framework and content of the questionnaire; the scale dimensions and item pool were also established. The KAP questionnaire regarding critically ill children with ICU-AW was evaluated by 15 experts (A total of 15 critical disease pediatricians, critical disease experts and nursing specialists in Beijing, Guangdong Province, Guizhou Province, Shanghai and Shandong Province were invited to complete 2 rounds of consultation by letter. There were 4 critical disease pediatricians, 6 pediatric critical disease nursing specialists, 3 medical specialists for critical diseases and 2 critical disease nursing specialists who were aged 40–58 and had worked for 15–36 years. In terms of the degree of education, there were 9 postgraduates and 6 doctoral students. There were 8 deputy senior titles and 7 senior titles), The total content validity index (CVI) of this scale was 0.923. The CVI of the 3 dimensions of this scale was 0.92, 0.87 and 0.91, respectively. The final questionnaire had 31 items and 3 dimensions (knowledge, attitudes, and practices) by Delphi expert consultation. The dimension on knowledge focused on the concept of ICU-AW, evaluation tools, clinical symptoms, sources, and staging. Each item was scored from 1 (not understood) to 3 (very well understood). The knowledge section contained 15 items with a total score of 45 points. The dimension on attitudes focused on PICU healthcare providers’ beliefs on perceptions and handling of the ICU-AW for critically ill children. Each item was scored on a scale of 1 (strongly disagree) to 5 (strongly agree). The dimension consisted of 8 items with a total score of 40. The dimension on practices contained the training, treatment, assessment, and management of PICU-AW-related knowledge by PICU health workers. Each item was scored on a scale of 1 (never) to 5 (always). The dimension contained 8 items with a total score of 40. Exploratory factor analysis was used to analyze the structural validity of the questionnaire. Bartlett’s test of sphericity and the Kaiser-Meyer-Olkin test were used to confirm that the measure was appropriate for exploratory factor analysis (Bartlett’s test of sphericity = 5566.07, Kaiser-Meyer-Olkin value = 0.87; P < 0.001) (See Table 3). A three-factor structure was yielded, which accounted for 59.49% of the total variance, indicating that the structure was consistent with the theoretical hypothesis. The Cronbach’s alpha coefficient of the questionnaire content, retesting reliability coefficient, and Spearman-Brown split-half value were 0.921, 0.888–0.909, and 0.967, respectively. The Cronbach’s alpha coefficient of knowledge ,Attitude,Practice were 0.92, 0.89,0.92.

**Table 3:** Response to statements regarding attitude of PICU Chinese Medical Staff on Children with ICU-AW (n%)

Attitude Statement	Mean ± SD	Strongly disagree	Disagree	Generally agree	Comparatively agree	Strongly agree
1. Do you agree that your knowledge of ICU-AW needs to meet clinical needs?	3.58 ± 1.061	19(3.6)	35(6.6)	236(44.5)	99(18.7)	141(26.6)
2. Do you think the PICU medical staff should observe the patient’s ICU-AW status dynamically like adults?	3.97 ± 0.907	3(0.6)	1(0.2)	205(38.7)	121(22.8)	200(37.7)
3. Do you think PICU medical staff should receive formal ICU-AW training?	4.07 ± 0.877	0	1(0.2)	184(34.7)	124(23.4)	221(41.7)
4. Do you think ICU-AW should be assessed as seriously as other complications (pressure ulcers, infections, etc.)?	4.04 ± 0.889	0	5(0.9)	184(34.7)	126(23.8)	215(40.6)
5. Do you think early functional exercise is very important for the prevention and recovery of ICU-AW?	4.06 ± 0.852	0	0	178(33.6)	144(27.2)	208(39.2)
6. Do you think healthcare workers should focus on ICU-AW prevention as much as other symptoms (e.g., delirium)?	4.00 ± 0.877	1(0.2)	9(1.7)	171(32.3)	158(29.8)	191(36.0)
7. Do you think it is the nurses and not others (doctors, technicians) who should assess the muscle strength of the child?	3.04 ± 1.094	53(10.0)	83(15.7)	250(47.2)	79(14.9)	65(12.3)
8. Do you think the ICU-AW status of critically ill patients should be included in the handover content of clinical work?	3.72 ± 0.955	7(1.3)	30(5.7)	203(38.3)	153(28.9)	137(25.8)

### Procedure

Due to the impact of COVID-19, the local government has implemented mandatory geographical isolation, closed the city (any form of traffic), and prohibited the movement of people.an online questionnaire platform—Sojump—was used to conduct the survey. The questionnaires were imported to Sojump and distributed by the platform. To control the quality of all questionnaires, the PICU manager of each hospital was contacted, and working groups were established for each. All individuals who answered the questionnaire were informed about the purpose of the study, investigators, risks, and benefits related to their participation. Those who completed the questionnaire received an electronic copy of the informed consent form before completing it. If they agreed to participate, they were automatically redirected to the questionnaire screen. Otherwise, they would automatically exit the questionnaire platform. Mandatory responses were established for all items to ensure the integrity of the questionnaire. Once the questionnaire was completed, the researcher exported the data from the web platform and stored the data securely.

### Data analysis

All information in the questionnaire was imported into Excel based on the sequence number. Statistical analysis was performed using SPSS 22.0 (IBM, Chicago, IL, United States). Frequencies, percentages, means, and standard deviations were used to present descriptive data, while means ± standard deviations and numbers (percentages) were used for continuous and categorical variables, respectively. Categorical variables were compared using t-tests and ANOVA, and multiple regression analysis was used to explore predictors of ICU-AW in critically ill children. For all statistical tests, two-tailed P-values < 0.05 were considered statistically significant.

### Ethical review

The study and consent procedure were approved by the ethics committee affiliated with ShenZhen people’s hospital (No:LL-KY-2022003-01). In addition,we confirmed that all methods were performed in accordance with the relevant guidelines and regulations.

## Results

### Demographic characteristics

A total of 530 questionnaires were distributed, and 530 valid questionnaires were received (100%). The response rate for this questionnaire is 100%.Among the participants, 51 (9.6%) were male and 479 (90.4%) were female; 428 (80.8%) were nurses and 102 (19.2%) were physicians. No rehabilitation therapists or respiratory therapists completed the questionnaire. Of the respondents, 369 (69.6%) were clinical nurses and 87 (16.5%) were clinicians; 401 (75.7%) were university bachelor’s degree holders, 54 (10.2%) had a master’s degree, and 10 (1.9%) had a doctoral degree. Most respondents were from Level 3 hospitals, with only 2.1% from Level 2 hospitals .The General Characteristics of participants in Table 4.

**Table 4:** Response to statements regarding practice of PICU Chinese Medical Staff on Children with ICU-AW (n%)

Practice Statement	Mean ± SD	Never	Sometimes	Usually	Always	Continuous
1. Do you actively pay attention to the patient’s ICU-AW status in your clinical work?	3.42 ± 1.011	32(6.0)	25(4.7)	249(47.0)	139(26.2)	85(16)
2. Do you communicate with patients about limb muscle strength in your clinical work?	3.36 ± 1.051	42(7.9)	32(6.0)	221(41.7)	162(30.6)	73(13.8)
3. Do you evaluate children’s ICU-AW in your clinical work?	2.85 ± 1.158	99(18.7)	65(12.3)	222(41.9)	105(19.8)	39(7.4)
4. Will you report the patient’s muscle strength to the doctor in the department timely?	3.57 ± 0.974	27(5.1)	20(3.8)	194(36.6)	204(38.5)	85(16.0)
5. Will you provide effective early functional exercise and dynamic assessment for critically ill children?	3.44 ± 1.007	31(5.8)	33(6.2)	214(40.4)	175(33.0)	77(14.5)
6. Will you instruct family members to help patients with appropriate activities to relieve symptoms such as physical weakness?	3.50 ± 1.018	35(6.6)	23(4.3)	193(36.4)	199(37.5)	80(15.1)
7. Do you make timely evaluations of nursing interventions for patients’ early mobilization?	3.34 ± 1.057	46(8.7)	34(6.4)	208(39.2)	178(33.6)	64(12.1)
8.Do you actively learn the relevant knowledge of ICU-AW at work?	3.06 ± 1.132	77(14.5)	44(8.3)	225(42.5)	136(25.7)	48(9.1)

### Dimensions of KAP regarding children with ICU-AW

The mean KAP scores of PICU health care workers towards critically ill children with ICU-AW were 30.35 ± 6.317,30.46 ± 5.632 and 26.54 ± 6.454, respectively. In the knowledge dimension, Q3 (Do you know how to diagnose ICU-AW?), Q4 (Do you know how to evaluate ICU-AW patients?), Q5 (Do you know how to evaluate ICU-AW patients?), and Q6 (Do you know the preventive measures of ICU-AW patients?) had lower mean scores. Only 20.4% were aware of the concepts related to ICU-AW, and only 23% of healthcare workers were aware that ICU-AW occurs in children as well as in adults. More than 50% of healthcare workers knew that ICU-AW increases the medical costs and mortality of children in the PICU.

The regarding knowledge of PICU children with ICU-AW in Table 5.

**Table 5:** Knowledge, Attitudes and Practice Levels of Chinese Medical Staff on Children with ICU-AW (Mean ± SD)

Variables	Knowledge Score	Attitude Score	Practice Score	Overall Score
**Gender**				
Male	31.92 ± 6.962	31.63 ± 4.472	29.06 ± 6.071	92.61 ± 13.793
Female	30.18 ± 6.229	30.34 ± 5.731	26.27 ± 6.441	86.80 ± 14.187
***t***	1.88	1.90	2.95	2.79
***P*** ^***a***^	0.061	0,062	0.003	0.006
**Age**				
18–25	30.0 ± 0 6.06	30.51 ± 5.906	27.21 ± 6.037	87.72 ± 14.337
26–30	29.71 ± 6.045	29.61 ± 5.467	25.92 ± 6.438	85.27 ± 14.489
31–40	30.83 ± 6.329	30.50 ± 5.662	26.42 ± 6.588	87.75 ± 13.496
41–50	29.71 ± 6.685	31.44 ± 5.388	27.36 ± 6.219	88.51 ± 15.426
51–60	33.43 ± 8.055	34.71 ± 4.065	28.14 ± 7.430	96.29 ± 15.862
***F***	1.76	3.319	1.009	2.34
***P*** ^***a***^	0.136	0.011	0.402	0.054
**Position**				
Doctor	31.33 ± 7.511	31.95 ± 4.802	26.01 ± 7.768	89.29 ± 14.680
Nurses	30.11 ± 5.984	30.11 ± 5.760	26.67 ± 6.102	86.90 ± 14.112
***t***	1.76	2.97	-0.92	1.53
***P*** ^***a***^	0.079	0.03	0.357	0.127
**Professional TiTle**				
Level 1	30.15 ± 6.128	30.01 ± 5.572	26.82 ± 6.444	86.98 ± 14.218
Level 2	30.21 ± 5.941	29.93 ± 5.410	26.41 ± 6.492	86.57 ± 14.067
Level 3	30.35 ± 6.363	30.67 ± 5.959	26.33 ± 6.396	87.35 ± 14.105
Level 4	30.03 ± 7.721	31.08 ± 4.709	26.30 ± 6.753	87.40 ± 15.130
Level 5	34.92 ± 6.739	36.69 ± 2.359	29.62 ± 5.994	101.2 ± 10.199
***F***	1.791	4.881	0.874	3.294
***P*** ^***a***^	0.129	＜0.001	0.479	0.011
**Position Title**				
physician manager	35.70 ± 7.732	34.60 ± 3.950	29.90 ± 3.071	100.2 ± 13.096
nurse manager	30.70 ± 5.856	29.59 ± 5.415	24.70 ± 4.195	85.00 ± 12.203
Advanced Practice Nurse	31.47 ± 7.558	30.87 ± 5.842	26.80 ± 5.414	89.13 ± 16.102
Clinical Teaching Supervisor	30.62 ± 10.445	32.00 ± 5.888	26.62 ± 3.380	89.23 ± 15.034
Nurses	30.12 ± 5.921	30.21 ± 5.779	26.82 ± 6.337	87.17 ± 14.268
Doctor	30.05 ± 6.727	31.41 ± 4.836	25.53 ± 8.169	86.99 ± 13.975
Research assistant	45.00 ± 0.000	36.00 ± 0.000	32.00 ± 0.000	113.00 ± 0.000
other	30.00 ± 1.414	24.00 ± 0.000	24.29 ± 0.756	78.29 ± 0.756
***F***	2.811	3.152	1.443	2.745
***P*** ^***a***^	0.007	0.003	0.185	0.008
**Education**				
Junior College	30.03 ± 6.119	28.80 ± 4.761	26.02 ± 6.753	84.85 ± 13.675
Undergraduate	30.14 ± 6.238	30.39 ± 5.742	26.85 ± 6.093	87.39 ± 14.334
Master	32.06 ± 6.284	32.78 ± 5.179	24.93 ± 8.335	89.76 ± 14.227
Doctor	31.40 ± 9.823	31.50 ± 5.104	26.40 ± 6.535	89.30 ± 13.491
***F***	1.610	5.183	1.574	1.251
***P*** ^***a***^	0.186	0.002	0.194	0.291
**Years of work**				
＜3	28.46 ± 5.773	28.99 ± 5.840	25.39 ± 5.822	82.83 ± 14.041
3–5	32.12 ± 6.148	31.21 ± 5.175	27.38 ± 6.484	90.71 ± 13.652
5–10	30.69 ± 6.574	30.22 ± 5.860	26.47 ± 6.373	87.41 ± 15.082
11–15	29.24 ± 5.541	30.37 ± 5.529	25.99 ± 7.068	85.60 ± 12.270
16–20	31.86 ± 5.254	30.86 ± 4.697	28.19 ± 5.203	90.92 ± 11.119
＞20	30.72 ± 7.685	32.13 ± 5.626	27.25 ± 6.630	90.09 ± 16.522
***F***	3.796	2.283	1.480	3.513
***P*** ^***a***^	0.02	0.045	0.195	0.004
**City**				
Level 1	30.55 ± 6.517	30.42 ± 5.697	26.24 ± 6.811	87.22 ± 14.993
Level 2	30.08 ± 5.480	31.14 ± 5.321	26.88 ± 6.903	88.10 ± 12.907
Level 3	29.98 ± 6.479	29.91 ± 5.709	27.16 ± 4.520	87.05 ± 13.099
***F***	0.425	1.229	0.963	0.176
***P*** ^***a***^	0.654	0.293	0.382	0.839
**Bed Range of PICU**				
＜10	30.22 ± 6.046	28.89 ± 5.131	25.34 ± 6.533	84.45 ± 14.306
11–15	30.87 ± 6.840	31.69 ± 5.514	27.53 ± 6.819	90.13 ± 14.887
16–20	31.10 ± 6.010	30.73 ± 5.646	26.81 ± 5.943	88.64 ± 13.598
＞20	29.32 ± 6.197	30.55 ± 5.935	26.30 ± 6.483	86.17 ± 13.680
***F***	3.106	5.058	2.050	3.978
***P*** ^***a***^	0.015	＜0.001	0.086	0.003
**Hospital Type**				
Level 3 Grade A general hospitals	30.05 ± 6.442	29.95 ± 5.474	26.39 ± 5.890	101.3 ± 14.680
Level 3 Grade A Children’s hospitals	30.52 ± 5.717	31.16 ± 5.855	26.38 ± 7.227	88.05 ± 14.023
Level 3 Grade B general hospitals	26.33 ± 1.155	36.67 ± 5.774	30.67 ± 16.166	88.05 ± 14.023
Level 3 Grade B Children’s hospitals	38.11 ± 5.667	33.89 ± 3.655	29.33 ± 8.411	86.40 ± 13.975
Level 2 Grade A Children’s hospitals	32.64 ± 6.667	34.18 ± 5.759	30.36 ± 8.201	97.18 ± 15.276
***F***	4.394	4.348	1.779	4.219
***P*** ^***a***^	0.002	0.002	0.132	0.002

In the attitude dimension with ICU-AW in Table 6, more than 80% of healthcare workers believed that training related to ICU-AW is needed, and more than 90% of PICU staff believed that ICU-AW should be given the same importance as other complications (e.g., delirium, pressure ulcers, unplanned extubation). All PICU healthcare workers believed that exercise rehabilitation should be actively used to prevent ICU-AW effectively. However, the scores for Q1 (Do you think your ICU-AW-related knowledge must meet clinical needs?) and Q7 (Did you know that critically ill children could also develop ICU-AW?) were low. In the practice dimension with ICU-AW in Table 7,Q3 (Do you evaluate children’s ICU-AW in your clinical work?), Q7 (Do you evaluate the patient’s early activity after nursing intervention promptly?), and Q8 (Do you actively seek relevant knowledge regarding ICU-AW during work?) had lower scores. More than 50% of PICU healthcare workers felt that they would report the child’s muscle strength during clinical work and would actively instruct the family to help the child with appropriate exercises to relieve the symptoms of muscle paralysis. However, only 34.8% of PICU healthcare workers would take the initiative to learn about ICU-AW .

**Table 6:** Associated factors of overall score of KAP regarding Chinese Medical Staff on Children with ICU-AW.

Variables	*B*	*SE*	*β*	*t*	*P*
Constant	3.011	0.332	—	9.076	＜0.001
Gender	-0.166	0.072	-0.106	-2.296	0.022
Age	-0.049	0.048	-0.101	-1.029	0.304
Position	0.005	0.068	0.004	0.069	0.945
Professional TiTle	0.027	0.035	0.057	0.758	0.449
Position Title	-0.022	0.021	-0.053	-1.069	0.286
Education	0.079	0.042	0.094	1.856	0.064
Years of work	0.036	0.029	0.111	1.228	0.220
City	0.028	0.027	0.048	1.053	0.293
Hospital Type	0.103	0.027	0.174	3.778	0.000
Bed Range of PICU	-0.017	0.017	-0.045	-1.001	0.317

Note:Adjusted R^2^ = 0.402 F = 2.994,*P*＜0.001

**Table 7:** Chinese medical staff professionals’hierarchies designations

Level	Description
Level 1	Nurse: Has a high school diploma and 3 years of working experience or a bachelor’s degree.
Level 2	Nurse Practitioner/Clinical Resident: Has a high school diploma and 5 years of service or a bachelor’s degree and 1 year of service.
Level 3	Supervising Nurse/Supervising Physician: Has a bachelor’s degree and 5 years of service or a postgraduate degree and 2 years of service.
Level 4	Associate Chief Nurse/Associate Chief Physician: Has a bachelor’s degree and 10 years of service or a doctoral degree and 3 years of service.
Level 5	Chief Nurse/Chief Physician: Has a doctoral degree, 5 years of service, and has published academic papers in related disciplines.

### Overall evaluation of KAP regarding children with ICU-AW

The mean total KAP score of PICU healthcare workers regarding critically ill children with ICU-AW was 87.36 ± 14.241. The overall score of KAP regarding children with ICU-AW using normality test showed a normal distribution, with scores ranging from 53 to 121. The overall KAP score regarding children with ICU-AW was ranked as poor (< 87, 50.56%), average (87–116, 46.04%), and good (> 116, 3.40%). The results from univariate analysis and the independent samples t-test showed large differences (*p* < 0.05) between gender, title, position, job title, years of service, number of beds, and hospital level. Total KAP scores for males were higher than females, and scores for positive senior titles were higher than for other titles (*p* < 0.05). Clinical directors, research assistants, and senior charge nurses in the department had higher KAP scores than those in other positions (*P* < 0.05). PICU personnel who had 16–20 years of experience had the highest total KAP scores (*P* < 0.05). Workers in PICUs with 11–15 beds in the hospital had higher total KAP scores than those in other PICUs, while workers in Level 3 general hospitals had higher KAP scores than those in hospitals from other categories (*p* < 0.05). Although there was no difference in total KAP scores between physicians and nurses, physicians had higher total KAP scores than nurses, where differences were seen in the attitude dimension (*p* < 0.05). The educational level also did not exhibit significant differences; however, the higher the level of education, the higher the total KAP score. Total KAP scores also increased gradually with the age of the respondents; more specifically, the attitude score towards ICU-AW increased gradually with the age of the respondents (*P* < 0.05), Levels of knowledge, attitude and practice regarding children with ICU-AW in Table 8. The results of multiple regression showed that gender, education, and hospital level were predictors of KAP level of PICU healthcare staff regarding critically ill children with ICU-AW .The Associated factors of overall score of KAP regarding children with ICU-AW in Table 9.

**Table 8:** The classification standard of Cinese hospitals

Hospital Level	Hospital Type	Description
**Level 1**	Primary Health Care Institution	Provides basic health care services
**Level 2**	Regional Hospital	Provides medical and health services across several communities. Most Level 2 hospitals in China do not have PICUs.
**Level 3**	Medical Prevention Technology Center	Provides medical and health services across regions, provinces, municipalities, and the whole country. These hospitals have comprehensive medical, teaching, and scientific research capabilities.
	Level 3 Grade A General Hospital	A type of Level 3 hospital with high-level facilities and equipment.
	Level 3 Grade A Children’s Hospital	A type of Level 3 hospital that focuses on providing medical care for children.
	Level 3 Grade B General Hospital	A type of Level 3 hospital with lower-level facilities and equipment compared to Level 3 Grade A hospitals.
	Level 3 Grade B Children’s Hospital	A type of Level 3 hospital that provides medical care for children with lower-level facilities and equipment compared to Level 3 Grade A children’s hospitals.

**Table 9:** Exploratory factor analysis of KAP questionnaire for critically ill children with ICU-AW

Variables	Factor-Loading		
	Knowledge	Attitude	Practice
A1.Do you know the related concepts of ICU-AW?	0.81		
A2.Do you know the clinical manifestations of ICU-AW?	0.87		
A3.Do you know how to diagnose ICU-AW?	0.82		
A4.Do you know how to evaluate ICU-AW patients?	0.86		
A5.Do you know the risk factors for ICU-AW?	0.87		
A6.Do you know the preventive measures of ICU-AW?	0.91		
A7.Did you know that critically ill children could also develop ICU-AW?	0.85		
A8.ICU-AW symptoms are muscle weakness with no clear cause in critically ill patients, clinically manifested as difficulty in weaning, paresis or quadriplegia, decreased reflexes, and muscle atrophy.	0.55		
A9.ICU-AW includes polyneuropathy in critically ill patients, myopathy in critically ill patients, and critical neuromuscular diseases.	0.73		
A10.The diagnosis of ICU-AW is mainly determined by the Medical Research Council Score (MRC-score).	0.70		
A11.Does the MRC-score use the Oxford Muscle Strength Scale to evaluate the six major muscle groups of the body?	0.84		
A12.ICU-AW not only prolongs the hospital stay and increases medical costs, but also reduces the patient’s ability to live and survive.	0.65		
A13. Braking may be an important risk factor for ICU-AW.	0.58		
A14. Early mobilization of ICU patients is the most effective intervention to prevent or mitigate ICU-AW in patients.	0.69		
A15. Standard insulin therapy can reduce the incidence and duration of neuromuscular complications, thereby reducing ICU-AW.	0.74		
B1. Do you agree that your knowledge of ICU-AW needs to meet clinical needs?		0.53	
B2. Do you think the PICU medical staff should observe the patient’s ICU-AW status dynamically like adults?		0.86	
B3. Do you think PICU medical staff should receive formal ICU-AW training?		0.90	
B4. Do you think ICU-AW should be assessed as seriously as other complications (pressure ulcers, infections, etc.)?		0.91	
B5. Do you think early functional exercise is very important for the prevention and recovery of ICU-AW?		0.87	
B6. Do you think healthcare workers should focus on ICU-AW prevention as much as other symptoms (e.g., delirium)?		0.90	
B7. Do you think it is the nurses and not others (doctors, technicians) who should assess the muscle strength of the child?		0.76	
B8. Do you think the ICU-AW status of critically ill patients should be included in the handover content of clinical work?		0.77	
C1. Do you actively pay attention to the patient’s ICU-AW status in your clinical work?			0.61
C2. Do you communicate with patients about limb muscle strength in your clinical work?			0.74
C3. Do you evaluate children’s ICU-AW in your clinical work?			0.53
C4. Will you report the patient’s muscle strength to the doctor in the department timely?			0.76
C5. Will you provide effective early functional exercise and dynamic assessment for critically ill children?			0.83
C6. Will you instruct family members to help patients with appropriate activities to relieve symptoms such as physical weakness?			0.80
C7. Do you make timely evaluations of nursing interventions for patients’ early mobilization?			0.77
C8.Do you actively learn the relevant knowledge of ICU-AW at work?			0.62
Eigenvalue explained for variance (%)	21.81	14.91	10.91
Cumulative variance(%)	21.81	42.02	59.49
Kaiser-Meyer-Olkin(KMO),Bartlett’s test of sphericity(χ^2^)	KMO = 0.87, χ^2^=5566.07		

## Discussion

The results indicated that the PICU healthcare staff had a moderate level of KAP regarding ICU-AW (87.36 ± 14.241).ICU-AW has been recognized as a complication in adults, of which CIM and CIN are important causes. ICU-AW is associated with many diseases in adults, including asthma, sepsis, and systemic inflammatory response syndrome. Our team has reviewed the literature on ICU-AW in adults [16]. Studies [17] have confirmed that more than 50% of adult patients experience varying degrees of ICU-AW, but ICU-AW is less explored among critically ill children due to the limited number of pediatric cases. This hinders the knowledge of PICU medical staff regarding ICU-AW (30.35 ± 6.317). The overall knowledge level of KAP regarding children with ICU-AW was moderate. Only 7.2%, 8.1%, and 11.5% of PICU medical staff knew about the diagnosis, assessment methods, and risk factors of ICU-AW. Aida Field-Ridley et al. [18] compared the differences in clinical outcomes between adults and children with ICU-AW. Their multivariate analysis showed that—similar to adults—mechanical ventilation, extracorporeal circulation, tracheotomy, and respiratory disease were risk factors for ICU-AW in pediatric populations.However, after reviewing 203,875 cases, the team had only 55 cases with ICU-AW; this is inconsistent with other studies [8]. The diagnosis of ICU-AW is strictly clinical and is usually assessed by manual muscle testing or hand-held dynamometry in adults. The Medical Research Council (MRC) is used to evaluate and diagnose ICU-AW in adult ICU patients [19] but is difficult to apply in critically ill children. The MRC-Score requires the patient’s cooperation during the assessment process and is susceptible to the assessor’s subjective consciousness during the scoring process. Therefore, PICU medical staff cannot easily detect if a critically ill child has ICU-AW. Consequently, the relatively poor level of knowledge directly affects the ability of PICU medical staff to act.

Assessment barriers are likely responsible for the inferior level of behavior (26.54 ± 6.454) of the ICU-AW of the PICU medical staff. Standard procedures for assessing the neuromuscular function of children in the PICU and the appropriate level of functional exercise are lacking. Nurses in the PICU prefer to keep children in a more comfortable state to reduce the pain associated with the disease, especially when a child is mechanically ventilated. Further, children are reluctant to cooperate with rehabilitation exercises due to discomfort, and their families are reluctant to undertake the risks of rehabilitation. Consequently, ICU-AW is usually detected among children in the PICU after withdrawal of mechanical ventilation.

Thankfully, almost all survey respondents indicated a willingness to receive training related to ICU-AW. All medical staff affirmed the benefits of rehabilitation exercises, and 99.1% of the PICU medical staff believed that ICU-AW should be treated at par with complications such as pressure ulcers and infections, which confirmed results from other studies [20]. However, who should perform the assessment remains debatable. Given that nurses have the most contact with patients, 74.4% of participants believed that nurses should assess the patient, since increased contact enhances the convenience of evaluation. However, neuromuscular assessment is performed diversely. In addition to the physical function, patients’ respiratory function, cough reflex, and nutritional status must be assessed. Considering this, a single assessment of muscle strength is inadequate to observe the status of the PICU child suffering from ICU-AW. A joint evaluation in collaboration with other medical personnel is required.

The results from the univariate analysis and independent samples t-test showed large differences (*p* < 0.05) between gender, title, position, job title, years of experience, number of beds, and hospital level.Evidence [21] suggests that highly educated people are more inquisitive compared to others, more adept at predicting the precursors of defending the conditions of critically ill children, and analyzing and summarizing the various problems arising in clinical work. Further, individuals with high education levels also have a good level of scientific thinking ability to access relevant information, as well as understand and seek solutions. The total KAP scores were also relatively higher for personnel with 16–20 years of experience and those with a senior job title. This may be because, along with the increase in years of service of PICU nurses, rank elevates due to their greater focus on improving their comprehensive ability and increasing level of work responsibility [22]. Long-term practical work broadens and enrichens knowledge related to critical care and the ability to explore difficult problems. Moreover, they are more willing to take the initiative to care for children in the PICU compared to other personnel [23].

The results of the multiple linear regression analysis showed that gender, education level, and hospital level were predictors of the KAP level of PICU healthcare workers regarding critically ill children with ICU-AW. The education level of PICU healthcare workers somewhat influenced the KAP scores of ICU-AW. Currently, ICU-AW is included neither in the content of Emergency and Critical Care Medicine or Emergency and Critical Care Nursing in Chinese medical schools nor in the continuing education training of new PICU employees. The concepts of ICU-AW and its research advances are only available in academic conferences, research reports, and journal forums. Therefore, it is suggested that managers include ICU-AW in future training and provide PICU staff with more opportunities for external study and visits, case discussions, and academic conferences to expand their perspective and improve their professionalism. In the present study, only 9.6% of the survey respondents were male, predicting that male staff would be better at assessing and preventing ICU-AW when faced with high-pressure, high-intensity, and high-risk PICU work.In China, PICUs require a certain level of hospital qualification and are mostly established in Level 3 hospitals. Only one PICU in this study was a level 2 hospital, thus a higher hospital level predicts a better level of KAP regarding ICU-AW.

The influence of ICU-AW knowledge, attitudes, and behaviors of PICU medical staff on critically ill children is a topic of international concern. ICU-AW is a common complication of critical illness in children worldwide, and its prevention and management are crucial to improving patient outcomes and reducing healthcare costs.Access to high-quality critical care services and trained PICU nurses can vary widely between countries. Developing countries may have limited resources and inadequate training for their PICU nurses, leading to higher rates of ICU-AW and poor patient outcomes.and Attitudes and beliefs regarding physical therapy and early mobilization may vary across different cultures, which could impact the willingness of PICU nurses to implement prevention and treatment strategies for ICU-AW [24] and Kim, J. S [25] provides a global perspective on the implementation of early rehabilitation programs in pediatric critical care medicine and highlights the role of PICU medical staff in these programs.the prevention and management of ICU-AW in critically ill children require the collaboration and coordination of healthcare professionals and systems worldwide. Medical staff play a critical role in this process, and their knowledge, attitudes, and behaviors are essential to providing high-quality care to critically ill children, regardless of their location or culture.By improving their knowledge and attitudes regarding the prevention and treatment of ICU-AW, PICU medical staff can provide better care to critically ill children and reduce the incidence and severity of ICU-AW. This can result in shorter ICU and hospital stays, reduced healthcare costs, and improved long-term outcomes for children and their families.In addition, This study that examine the influence of ICU-AW knowledge, attitudes, and behaviors of PICU nurses on critically ill children can also highlight the importance of early rehabilitation and physical therapy interventions in promoting physical function and preventing long-term disability.Ultimately, by promoting better medical practices in the prevention and management of ICU-AW, critically ill children can receive high-quality, evidence-based care that promotes their physical function and overall well-being.

This study has some limitations. First, the sample size was small. Although 38 hospitals responded, only 13 respondents on average came from each region. This is also likely related to the setup of PICUs in China. Most PICUs in the present study had around 10 beds, thus there were less personnel in the PICU. Second, the respondents in this study were mainly doctors and nurses, although some became research assistants and managers. There were no responses from rehabilitation therapists, psychotherapists, or other professionals. This may limit the generalizability of the findings.Due to selection bias, nurses were the most positive group to respond to the survey. This is likely due to the fact that nurses make up the majority of medical staff in the PICU. Additionally, as the first point of contact for patients, nurses tend to have positive emotions towards their patients, making them the most likely group to respond to the survey. Finally, due to the impact of the COVID-19 epidemic in China, communications with survey respondents were restricted due to Chinese policy. Therefore, only online questionnaires could be conducted, which affected the quality of the survey to some extent.

## Conclusions

In summary, the results of this study showed that the medical staff of PICU had a moderate KAP level regarding children with ICU-AW. Total KAP scores were lower in females, junior nurses, staff with college diplomas, personnel with < 3 years of service, and those from Level 3 Grade B hospitals. Gender, education, and hospital level were predictors of the KAP status of PICU healthcare staff regarding critically ill children with ICU-AW. Therefore, PICU leaders need targeted acute training programs for PICU medical staff.

## Electronic supplementary material

Below is the link to the electronic supplementary material.

Supplementary Material 1

## Data Availability

The datasets generated and analysed during the current study are not publicly available due to the confidentiality agreement with the participants. The data is however available from the corresponding author on reasonable request.
