# Supplementary material for: Knowledge, attitudes and practices regarding children with ICU-acquired weakness in pediatric intensive care unit among chinese medical staff: a cross-sectional survey
Source: BMC Nurs. 2023 May 15;22:162. doi: 10.1186/s12912-023-01304-x (PMC10184079; doi:10.1186/s12912-023-01304-x)
Supplement: Supplementary file 1 — Supplementary Material 1 [file 12912_2023_1304_MOESM1_ESM.docx]

**Knowledge, Attitude, and Practice Questionnaire for PICU Children with ICU-AW**

**Dear PICU medical staff:**

Hello! Thank you very much for participating in this survey.

Based on the theory of knowledge, belief, and action, this study will investigate the knowledge, attitude, behavior status and influencing factors of PICU medical staff in major hospitals across the country on the assessment of ICU-acquired weakness in critically ill children (ICU-AW). The purpose of this study is to promote PICU medical staff's attention to ICU-AW in critically ill children, timely assessment of risk factors and early prevention. This survey is anonymous, therefore please fill it out truthfully and independently. The information you provide will be only used for scientific research and will not have any impact on your organization or individual work. At the same time, this survey will strictly abide by the principle of confidentiality. We sincerely thank you for your support and participation in this research! This research will take you 5-10 minutes.

**General Information**

1. Your gender: ①Male ②Female

2. Your age group: ①18-25; ②26-30;③31-40; ④41-50; ⑤51-60

3. Your job category in PICU is: ① doctor ② nurse

4. Your current job title is: ①assistant nurse (Level 1) ②Nurse (Level 2) ③Supervisor nurse/Chief Physician(Level 3) ④Deputy Chief Nurse/Deputy Chief Physician(Level 4) ⑤Chief Nurse/Chief Physician(Level 5)

5. Your current position is: ①Department Director ②Head Nurse ③Senior Practice Nurse ④Clinical Teaching Supervisor ⑤Clinical Nurse ⑥Clinician ⑦Research Assistant ⑧Others

6. Your education level is: ①Junior College ②Undergraduate ③Master ④Doctor

7. Your working years: ①＜3 ②3-5 ③5-10 ④11-15 ⑤16-20 ⑥＞20

8. Your city level: ①Tier 1 cities(Level 1) ②Tier 2 cities(Level 2) ③Tier3 cities(Level 3)

9. The level of the hospital you are in: ① Level 3 Grade A general hospitals②Level 3 Grade A Children's hospitals ③ Level 3 Grade B general hospitals④Level 3 Grade B Children's hospitals⑤ Level 2 Grade A general hospitals⑥ Level 2 Grade A Children's hospitals

10. What is the bed range of your PICU?

①＜10 ②11-15 ③16-20 ④＞20

**Knowledge, Attitude, and Practice Questionnaire**

1. **Knowledge**

1. Do you know the related concepts of ICU-AW?

①Do not know ②Generally understand ③Well understand

2. Do you know the clinical manifestations of ICU-AW?

①Do not know ②Generally understand ③Well understand

3. Do you know how to diagnose ICU-AW?

①Do not know ②Generally understand ③Well understand

4. Do you know how to evaluate ICU-AW patients?

①Do not know ②Generally understand ③Well understand

5. Do you know the risk factors for ICU-AW?

①Do not know ②Generally understand ③Well understand

6. Do you know the preventive measures of ICU-AW?

①Do not know ②Generally understand ③Well understand

7. Did you know that critically ill children could also develop ICU-AW?

①Do not know ②Generally understand ③Well understand

8. ICU-AW symptoms are muscle weakness with no clear cause in critically ill patients, clinically manifested as difficulty in weaning, paresis or quadriplegia, decreased reflexes, and muscle atrophy.

①Do not know ②Generally understand ③Well understand

9. ICU-AW includes polyneuropathy in critically ill patients, myopathy in critically ill patients, and critical neuromuscular diseases.

①Do not know ②Generally understand ③Well understand

10. The diagnosis of ICU-AW is mainly determined by the Medical Research Council Score (MRC-score).

①Do not know ②Generally understand ③Well understand

11. Does the MRC-score use the Oxford Muscle Strength Scale to evaluate the six major muscle groups of the body?

①Do not know ②Generally understand ③Well understand

12. ICU-AW not only prolongs the hospital stay and increases medical costs, but also reduces the patient's ability to live and survive.

①Do not know ②Generally understand ③Well understand

13. Braking may be an important risk factor for ICU-AW.

①Do not know ②Generally understand ③Well understand

14. Early mobilization of ICU patients is the most effective intervention to prevent or mitigate ICU-AW in patients.

①Do not know ②Generally understand ③Well understand

15. Standard insulin therapy can reduce the incidence and duration of neuromuscular complications, thereby reducing ICU-AW. ,

①Do not know ②Generally understand ③Well understand

1. **Attitude**

1. Do you agree that your knowledge of ICU-AW needs to meet clinical needs?

①Strongly disagree ②Disagree ③Generally agree ④Comparatively agree ⑤Strongly agree

2. Do you think the PICU medical staff should observe the patient's ICU-AW status dynamically like adults?

①Strongly disagree ②Disagree ③Generally agree ④Comparatively agree ⑤Strongly agree

3. Do you think PICU medical staff should receive formal ICU-AW training?

①Strongly disagree ②Disagree ③Generally agree ④Comparatively agree ⑤Strongly agree

4. Do you think ICU-AW should be assessed as seriously as other complications (pressure ulcers, infections, etc.)?

①Strongly disagree ②Disagree ③Generally agree ④Comparatively agree ⑤Strongly agree

5. Do you think early functional exercise is very important for the prevention and recovery of ICU-AW?

①Strongly disagree ②Disagree ③Generally agree ④Comparatively agree ⑤Strongly agree

6. Do you think healthcare workers should focus on ICU-AW prevention as much as other symptoms (eg, delirium)?

①Strongly disagree ②Disagree ③Generally agree ④Comparatively agree ⑤Strongly agree

7. Do you think it is the nurses and not others (doctors, technicians) who should assess the muscle strength of the child?

①Strongly disagree ②Disagree ③Generally agree ④Comparatively agree ⑤Strongly agree

8. Do you think the ICU-AW status of critically ill patients should be included in the handover content of clinical work?

①Strongly disagree ②Disagree ③Generally agree ④Comparatively agree ⑤Strongly agree

**3.Practice**

1. Do you actively pay attention to the patient's ICU-AW status in your clinical work?

①Never ②Sometimes③Usually④Always⑤Continuous

2. Do you communicate with patients about limb muscle strength in your clinical work?

①Never ②Sometimes③Usually④Always⑤Continuous

3. Do you evaluate children's ICU-AW in your clinical work?

①Never ②Sometimes③Usually④Always⑤Continuous

4. Will you report the patient's muscle strength to the doctor in the department timely?

①Never ②Sometimes③Usually④Always⑤Continuous

5. Will you provide effective early functional exercise and dynamic assessment for critically ill children?

①Never ②Sometimes③Usually④Always⑤Continuous

6. Will you instruct family members to help patients with appropriate activities to relieve symptoms such as physical weakness?

①Never ②Sometimes③Usually④Always⑤Continuous

7. Do you make timely evaluations of nursing interventions for patients' early mobilization?

①Never ②Sometimes③Usually④Always⑤Continuous

8. Do you actively learn the relevant knowledge of ICU-AW at work?

①Never ②Sometimes③Usually④Always⑤Continuous
